# Primary anorectal malignant melanoma: a rare but aggressive tumor: report of a case

**DOI:** 10.1186/s12957-014-0419-z

**Published:** 2015-01-30

**Authors:** Dominique Buissin, Alenka Sterle, Peter Schmiegelow, Dirk Wassenberg, Peter C Ambe

**Affiliations:** Institute of Pathology, Städtliches Klinik Solingen, Gotenstraße 1, Solingen, Germany; Department of General, Visceral and Thoracic Surgery, St. Remigius Hospital Opladen, An St. Remigius 26, 51379 Leverkusen, Germany; Helios Klinikum Wuppertal, Department of Surgery II, Witten – Herdecke University, Heusner Str. 40, 42283 Wuppertal, Germany

**Keywords:** Anorectal melanoma, Transanal polypectomy, Melan A, Abdominoperineal rectum resection, Wide local resection

## Abstract

This report presents a case of primary anorectal melanoma. A 63-year–old male presented with blood in stool. Rectal digital examination and proctoscopy revealed a mass in the anorectal junction. Transanal polypectomy was performed. Histopathology and immunohistochemistry with Melan A showed a malignant anorectal melanoma with positive resection margins. Abdomino-perineal rectum resection was performed after excluding distant metastasis. Four month later, the patient was readmitted with metastases to the liver and to the gastric mucosa. Best supportive care was initiated. This case report demonstrates the aggressive nature of this rare tumor and appeals for a less aggressive management while maintaining the quality of life.

## Background

Primary anorectal malignant melanoma is an extremely rare malignancy that is thought to arise from melanocytes in the mucosa around the anorectal junction. AMM is associated with a relatively poor prognosis, and surgical resection is the only curative treatment option. This case demonstrates the aggressive nature of this rare entity.

## Case presentation

**Figure 1:**
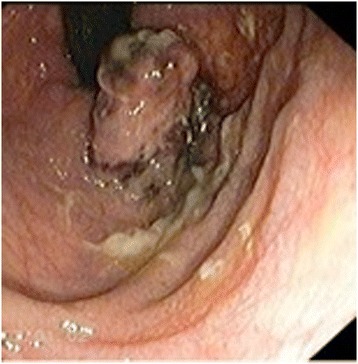
**Endoscopic finding.** Proctoscopy revealed a mass at the anorectal junction.

**Figure 2:**
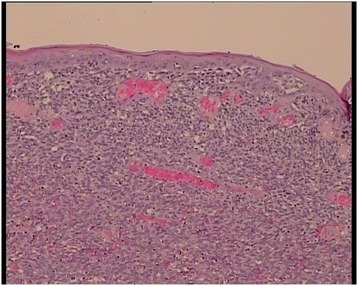
**Hematoxylin and eosin stain.** Infiltration of the mucosa through malignant cells.

**Figure 3:**
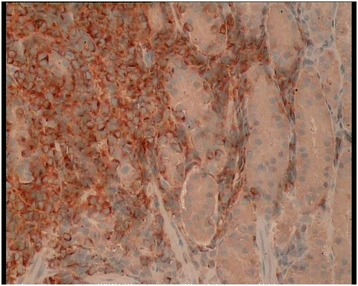
**Immunohistochemistry.** Immunohistochemical staining with Melan A confirmed the presence of melanocytes in the malignant cells.

**Figure 4:**
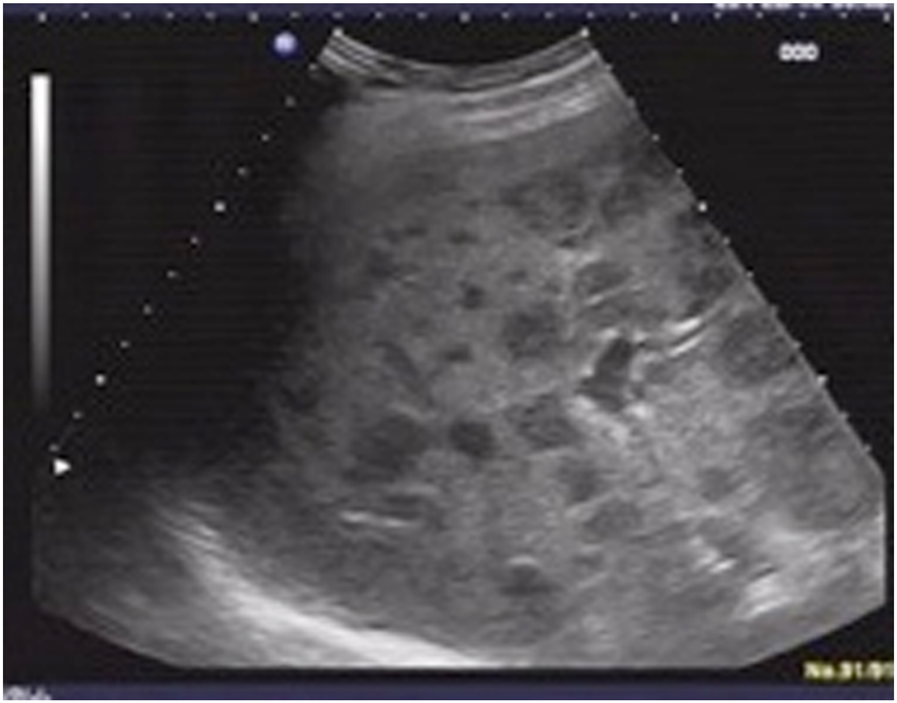
**Liver ultrasound.** Multiple liver lesions.

**Figure 5:**
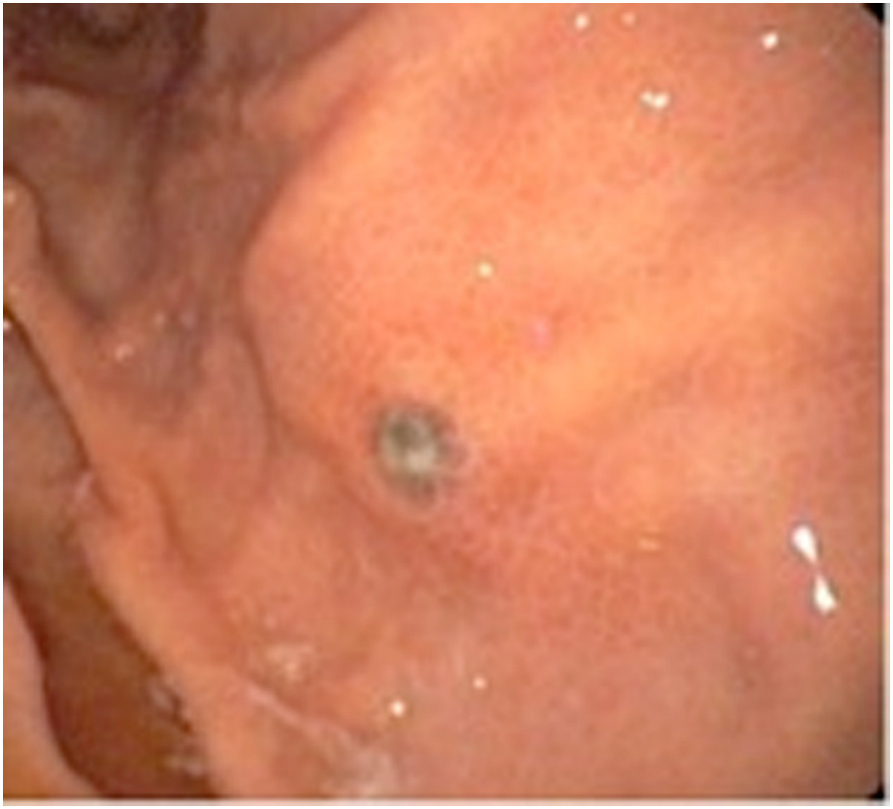
**Upper GI endoscopy.** Pigmented lesion in the gastric mucosa stained positive for Melan A.

A 63-year-old man presented with a history of blood in the stool. Rectal examination revealed a mass in the lower rectum. A broad-based rectal polyp about 6 cm from the anal verge was seen on proctoscopy, and biopsies were taken (Figure 1). Histopathology showed a hyperplastic polyp. A transanal polypectomy was performed after colonoscopy. Histopathology diagnosed a malignant melanoma (Figure 2) with positive stains for melan A on immunohistochemistry (Figure 3), with positive resection margins (pT4bR1). Other primary sites and distant metastases were excluded. The patient was managed with abdominoperineal resection (APR), following the consensus of the interdisciplinary tumor board. Surgery and recovery were uneventful. The final tumor stage was pT4bN0 (0/15) cM0pR0, stage IIC (UICC). Four months later, the patient was readmitted with tenderness in the right upper quadrant. Ultrasound demonstrated multiple metastatic lesions in the liver (Figure 4). Endoscopy revealed pigmented lesions in the gastric mucosa (Figure 5); Histopathology confirmed metastasis of a malignant melanoma. Best supportive care was initiated.

## Discussion

First reported by Moore DW in 1857 [1], AMM is an extremely rare malignancy that is thought to arise from melanocytes in the mucosa around the anorectal junction. AMM constitutes about 0.05% of all anorectal malignancies [2,3]. The rare nature of this entity is represented by the limited number of cases described in the medical literature. The largest series from a single center included 85 cases from the Memorial Sloan-Kettering Cancer Center, reported by Brady et al. in 1995 [3].

AMM is mostly seen in the sixth decade, with a female predominance [4,5], and rectal bleeding is the most common symptom. Changes in bowel movements, rectal pain, and inguinal mass may also be present. Hemorrhoids, polyps, and other malignancies are the most common differential diagnoses [3,4].

A mass is usually palpated on digital rectal examination. Proctoscopy usually reveals a hemorrhoid-like pigmented lesion near the anorectal junction, on which a biopsy must be performed. The histopathologic findings are similar to those of melanomas of other origins (that is. the identification of melanin and immunohistochemical staining for S100, HMD-45, and/or Melan A [6].

After the histologic diagnosis of AMM, a complete staging and search for possible distant metastases (colonoscopy, computed tomography of the abdomen and thorax, MRI of the pelvis and brain), as well as ruling out primary sites (skin and retina), are mandatory.

Each patient with AMM should be discussed in an interdisciplinary board, and treatment should be individualized based on the tumor size, age, comorbidities, and absence or presence of metastases.

Surgical resection is the mainstay of treatment [5,7-9]. APR with or without bilateral inguinal lymphadenectomy and wide local excision (WLE) have been used to manage patients with AMM. APR is thought to reduce the probability of recurrence by controlling the spread to mesenteric lymph nodes and creating a larger negative resection margin [3,4,9,10]. However, available data suggest no significant difference in survival among patients managed with APR and WLE. Because APR is associated with high rates of morbidity and colostomy-associated decrease in the quality of life, many authors advocate WLE if negative margins are achievable [7]. Chemotherapy and radiotherapy have no benefit [11].

The prognosis is poor, with overall survival rate <20% in 5 years. Age >60 years and lesions >1 cm in diameter have been identified as prognostic factors [3,4,8,9].

## Conclusion

In the case presented, AMM was diagnosed after transanal polypectomy with positive margins; thus APR was performed. Despite aggressive surgical management, disease progression was very rapid; with the development of hepatic and gastric metastases within 4 months. This case demonstrates the aggressive nature of AMM and questions the role of aggressive surgery (for example, APR) in the management of AMM. Therefore, until more evidence becomes available, the quality of life must be kept in focus when managing AMM, as is the case with WLE.

## Consent

A written informed consent was obtained from the patient for the publication of this case report with the accompanying images. A copy of the consent is available for review from the Editor-in-Chief of this journal.
